# Targeting TREM-1 Signaling in the Presence of Antibiotics is Effective Against Streptococcal Toxic-Shock-Like Syndrome (STSLS) Caused by *Streptococcus suis*

**DOI:** 10.3389/fcimb.2015.00079

**Published:** 2015-11-12

**Authors:** Chao Yang, Jianqing Zhao, Lan Lin, Shan Pan, Lei Fu, Li Han, Meilin Jin, Rui Zhou, Anding Zhang

**Affiliations:** ^1^National Key Laboratory of Agricultural Microbiology, Huazhong Agricultural UniversityWuhan, China; ^2^College of Veterinary Medicine, Huazhong Agricultural UniversityWuhan, China; ^3^The Cooperative Innovation Center for Sustainable Pig Production, Huazhong Agricultural UniversityWuhan, China; ^4^Key Laboratory of Development of Veterinary Diagnostic Products, Ministry of Agriculture, Huazhong Agricultural UniversityWuhan, China

**Keywords:** *Streptococcus suis* (*S. suis*), Streptococcal toxic-shock-like syndrome (STSLS), Triggering receptor expressed on myeloid cells-1 (TREM-1), anti-inflammatory treatment, treatments targets

## Abstract

*Streptococcus suis* (*S*.suis), a major swine pathogen, is also a severe threat to human health. Infection with highly virulent strains of *S. suis* can cause human Streptococcal toxic-shock-like syndrome (STSLS), which is associated with high serum pro-inflammatory cytokine levels and a high mortality rate. Our previous study indicated that highly virulent *S. suis* infection could activate the TREM-1 signaling pathway, which promotes host clearance of *S. suis* during early infection. However, it remained to be elicited whether TREM-1 signaling could be a target against STSLS in the presence of antibiotic. In the present study, mice were infected with a highly virulent *S. suis* strain and then treated with rTREM-1 (the recombinant extracellular domain of TREM-1) to block TREM-1 signaling, antibiotics, both rTREM-1 and antibiotics, or PBS. The survival rates, clinical signs, serum IL-1β and TNF-α levels, and serum bacterial loads were evaluated. Treatment with rTREM-1 could aggravate the outcome of infection as described previously. Although the conventional treatment with antibiotics contributed to effective *S. suis* clearance, it did not improve survival significantly. In comparison, due to the reduction of the exaggerated pro-inflammatory response, treatment combined with rTREM-1 and antibiotics not only led to efficient bacterial clearance but also alleviated inflammation. In conclusion, TREM-1 signaling contributed to severe inflammatory response and benefited *S. suis* clearance. Therefore, blocking TREM-1 signaling could still be a target for the treatment of STSLS in the presence of antibiotics.

## Introduction

*Streptococcus suis* (*S. suis*) is a major swine pathogen that is responsible for severe economic losses in the porcine industry, and it is also a severe threat to human health (Segura, 2009; Wertheim et al., 2009; Gottschalk et al., 2010b; Gottschalk, 2012). Since the first reported case of *S. suis*-induced meningitis in humans in Denmark in 1968, more than 1500 human infection cases have been reported in the world (Huong et al., 2014). In addition, *S. suis* has also been identified as the third most common cause of community-acquired bacterial meningitis in Hong Kong and as the leading and second cause of adult meningitis in Vietnam and in Thailand, respectively (Suankratay et al., 2004; Mai et al., 2008; Segura, 2009). *S. suis* infection in humans has remained sporadic for a long time and mainly affects individuals in close contact with sick or carrier pigs or pig-derived products (Huang et al., 2005; Smith et al., 2008; Fowler et al., 2013). However, the large-scale outbreaks in China in 1998 and 2005 (Tang et al., 2006; Yu et al., 2006) and a case of human meningitis without a history of animal contact (Manzin et al., 2008; Callejo et al., 2014) have modified perspectives regarding the threat of *S. suis* to humans. Furthermore, human *S. suis* isolates showed high degrees of adhesion to human intestinal epithelial cells, suggesting that the pathogen should be considered a food-borne pathogen (Ferrando et al., 2014). In addition, *S. suis* infection is very dangerous for malignancy patients (Gomez-Zorrilla et al., 2014) people with alcoholism (Nakayama et al., 2013) and splenectomy patients (Gallagher, 2001; Tambyah and Lee, 2001). It might also cause adverse clinical outcomes for influenza-threatened people (Wang and Lu, 2008; Dang et al., 2014).

*S. suis* infection in humans typically produces purulent or nonpurulent meningitis, endocarditis, cellulitis, peritonitis, rhabdomyolysis, arthritis, spondylodiscitis, pneumonia, uveitis, endophthalmitis, and occasionally septic shock (Segura, 2009; Choi et al., 2012; Zalas-Wiecek et al., 2013). Special attention should be given to the largest recorded outbreak in 2005 in China, in which 38 deaths were reported among 204 documented human cases. Of the 38 deaths, 37 were associated with septic shock, which was designated “Streptococcal toxic-shock-like syndrome (STSLS)” (Tang et al., 2006). Notably, 63% of STSLS patients died rapidly even after antimicrobial treatment (Lun et al., 2007) and this disease was characterized by high serum levels of IFN-γ, TNF-α, IL-8, IL-12, and IL-1β (Ye et al., 2009). Studies using experimentally infected mice have demonstrated that induction of high levels of systemic pro-inflammatory cytokines plays an important role in sudden death or meningitis (Lachance et al., 2014). In addition, the IFN-γ response was also considered to be responsible for the high mortality rate of STSLS (Lachance et al., 2013). These data suggested that inhibiting the exaggerated inflammatory response may benefit the outcome of the disease.

Triggering receptor expressed on myeloid cells-1 (TREM-1), which belongs to the Ig superfamily, is an activating receptor that is expressed on myeloid cells (Bouchon et al., 2000). It can be induced at high levels on neutrophils and monocytes and further amplifies Toll-like receptor (TLR)-initiated responses against microbial challenges, potentiating the secretion of proinflammatory cytokines with the help of the DAP12 adaptor protein in response to bacterial and fungal infections (Bouchon et al., 2001; Colonna and Facchetti, 2003; Dower et al., 2008). Due to the key role of TREM-1 in amplifying the inflammatory response, TREM-1 was identified as an essential regulator of innate immunity in sepsis syndrome (Nathan and Ding, 2001) and it was also confirmed to be an attractive target for the treatment of septic shock (Gibot et al., 2006, 2007), sepsis (Bouchon et al., 2001; Gibot et al., 2004; Wang et al., 2012; Pieters et al., 2013; Van Bremen et al., 2013), inflammatory bowel disease (Holden et al., 2009; Genua et al., 2014), chronic inflammatory disorders (Schenk et al., 2007), autoimmune arthritis (Murakami et al., 2009), corneal inflammation (Wu et al., 2011), and hepatocellular chronic inflammation (Wu et al., 2012).

Our previous transcriptional analysis on swine spleen cells in response to *S. suis* infection indicated that TREM-1 expression was up-regulated and that a few pro-inflammatory genes were highly expressed (Li et al., 2010). The TREM-1-mediated innate immune response was confirmed to play an essential role in the activation of neutrophils, which further benefited the outcome of the infection by improving *S. suis* clearance (Yang et al., 2015). However, the direct role of the TREM-1-mediated innate immune response on STSLS is still unknown. Therefore, the present study aimed to determine the contribution of this signaling on severe inflammation. Because the TREM-1-mediated innate immune response played a role during *S. suis* clearance, in the present study, the recombinant extracellular domain of TREM-1 (rTREM-1) was used to inhibit signaling in the presence of antibiotics to evaluate the direct role of TREM-1-mediated innate immune response on STSLS. In addition, the present study also aimed to determine whether TREM-1 signaling could be a target for treatment of STSLS in the presence of antimicrobial drugs.

## Materials and methods

### Bacterial strains

*S. suis* serotype 2 strain 05ZY (also known as SC-19) was isolated from the brain of a diseased piglet during the outbreak of *S. suis* diseases in China in 2005. The strain expresses muramidase-released protein, extracellular protein factor, and suilysin and is highly pathogenic to mice and pigs, causing STSLS (Zhang et al., 2011, 2012).

The bacteria were cultured for 12 h in Tryptone Soya Broth (BD) plus 10% bovine sera at 37°C to reach stationary phase. Then 500 μl of the bacteria suspension was added into 50 ml of fresh Tryptone Soya Broth plus 10% bovine sera and cultured for 4–6 h to reach log-phase. Then the bacteria suspension was placed on ice to stop growth and then washed with PBS for two times. Finally, the concentration of the bacteria suspension was adjusted to 8 × 10^8^ CFU/ml for animal experiments. CFU per ml values were further confirmed by spreading the serially diluted bacteria suspension on Tryptone Soya Agar (BD) plates.

### Preparation of rTREM-1

The recombinant extracellular domain of TREM-1 (rTREM-1) was prepared according to the previous procedure (Yang et al., 2015). Before rTREM-1 was used as the blocking agent, the protein was confirmed to inhibit the enhanced inflammatory response of platelets to LPS stimulation as described before (Yang et al., 2015).

### Experimental infection of mice

The study was performed in strict accordance with the Guide for the Care and Use of Laboratory Animals Monitoring Committee of Hubei Province, China, and the protocol was approved by the Committee on the Ethics of Animal Experiments at the College of Veterinary Medicine, Huazhong Agricultural University (Permit Number: 4200060000681). All efforts were made to minimize the suffering of the animals used in the study.

Forty 6-week-old female C57BL/6 mice with similar body weights were randomly divided into four groups with 10 mice in each group. All mice were inoculated with an intraperitoneal injection of 0.5 ml of a *S. suis* strain (05ZYS) suspension at 8 × 10^8^ CFU/ml. After 3 h, the four groups were treated by an intraperitoneal injection of 0.5 ml of PBS, ampicillin (2 mg/ml), rTREM-1(120 μg/ml), or a combination of ampicillin (2 mg/ml) and rTREM-1 (120 μg/ml). The mice were monitored three times per day for 5 days for clinical signs and were assigned clinical scores as described by Dominguez-Punaro et al. (2007). Mice exhibiting extreme lethargy or neurological signs were considered moribund and were humanely killed. The body weight of each mouse was recorded every day. At the end of the experiment, the surviving animals were sacrificed via carbon dioxide inhalation.

Sixty 6-week-old female C57BL/6 mice with similar body weights were randomly divided into five groups with 12 mice in each group. Groups 1–4 were infected by an intraperitoneal injection with 0.5 ml of a *S. suis* strain (05ZYS) suspension at 8 × 10^8^ CFU/ml, and group 5 was mock infected with PBS and served as a control. At 3 h post-infection, groups 1–4 were treated with an intraperitoneal injection of 0.5 ml of PBS, ampicillin (2 mg/ml), rTREM-1(120 μg/ml), or a combination of ampicillin (2 mg/ml) and rTREM-1(120 μg/ml). Group 5 was mock treated with PBS at that time point. At 3, 6, 9, and 24 h after infection, three mice in each group were sacrificed via carbon dioxide inhalation, and anticoagulated blood was collected via a cardiac puncture. Fifty microliters of anticoagulated blood was withdrawn for bacterial loading analysis. The remaining blood was used to prepare plasma for the analysis of the IL-1β and TNF-α concentrations.

### Bacteria load in blood

The collected blood samples were serially diluted and then plated on Tryptone Soya Agar (BD) plates to evaluate bacterial load.

### Cytokine measurements

The collected blood samples were serially diluted and then were used to analyze the IL-1β and TNF-α concentrations with ELISA kits according to the manufacturer's protocols (CityDakeweGroup, China). The samples and the known standards were assayed in triplicate.

### Histopathologic evaluation

The lungs of mice at 6 h post-infection were fixed in 10% neutral buffered formalin and routinely processed into paraffin. Sections 2 to 3 mm in thickness were cut for hematoxylin and eosin staining for histopathologic evaluation.

All lung samples were examined and scored by the technician in National Key Laboratory of Agricultural Microbiology who blinded to treatment and intervention. Five areas were randomly selected from the every histopathologic section and then were scored based on the severity and inflammation as following: 0 = Normal; 1 = Minimal hemorrhagic, consisting of occasional degenerate neutrophils with extravasated erythrocytes and fibrin and necrotic cellular debris; 2 = Mild hemorrhagic, consisting of low numbers of degenerate neutrophils with extravasated erythrocytes and fibrin and necrotic cellular debris; 3 = Moderate hemorrhagic, consisting of moderate numbers of degenerate neutrophils with extravasated erythrocytes and fibrin and necrotic cellular debris; 4 = Severe hemorrhagic, consisting of large numbers of degenerate neutrophils with abundant extravasated erythrocytes and fibrin and necrotic cellular debris.

### Statistical analysis

Unless otherwise specified, the data were analyzed using two-tailed, unpaired *t*-tests, and all assays were repeated at least three times. The data were expressed as the means ± standard deviations. For the animal infection experiments, survival was analyzed using the log-rank test in GraphPad Prism 5. A value of *p* < 0.05 was considered as the threshold for significance.

## Results and discussion

*S. suis* was recognized as a new emerging or old neglected zoonotic pathogen (Gottschalk et al., 2010a). The infection can induce high levels of inflammatory cytokines, an important character of STSLS (Ye et al., 2009) which attracted researchers to consider whether targeting the inflammatory response was effective against STSLS (Dominguez-Punaro Mde et al., 2008; Lagler et al., 2009; Lachance et al., 2013, 2014). Our previous study indicated that TREM-1 regulated the innate immune response and benefited clearance of *S. suis* (Yang et al., 2015). Although direct targeting TREM-1 signaling was not effective, it remained to be elicited whether the treatment was effective when bacteria propagation was under the control. Therefore, the present study used the recombinant extracellular domain of TREM-1 (rTREM-1) as an inhibitor of TREM-1 signaling to evaluate the direct contribution of TREM-1 signaling during STSLS in the presence of antibiotics.

**Figure 1:**
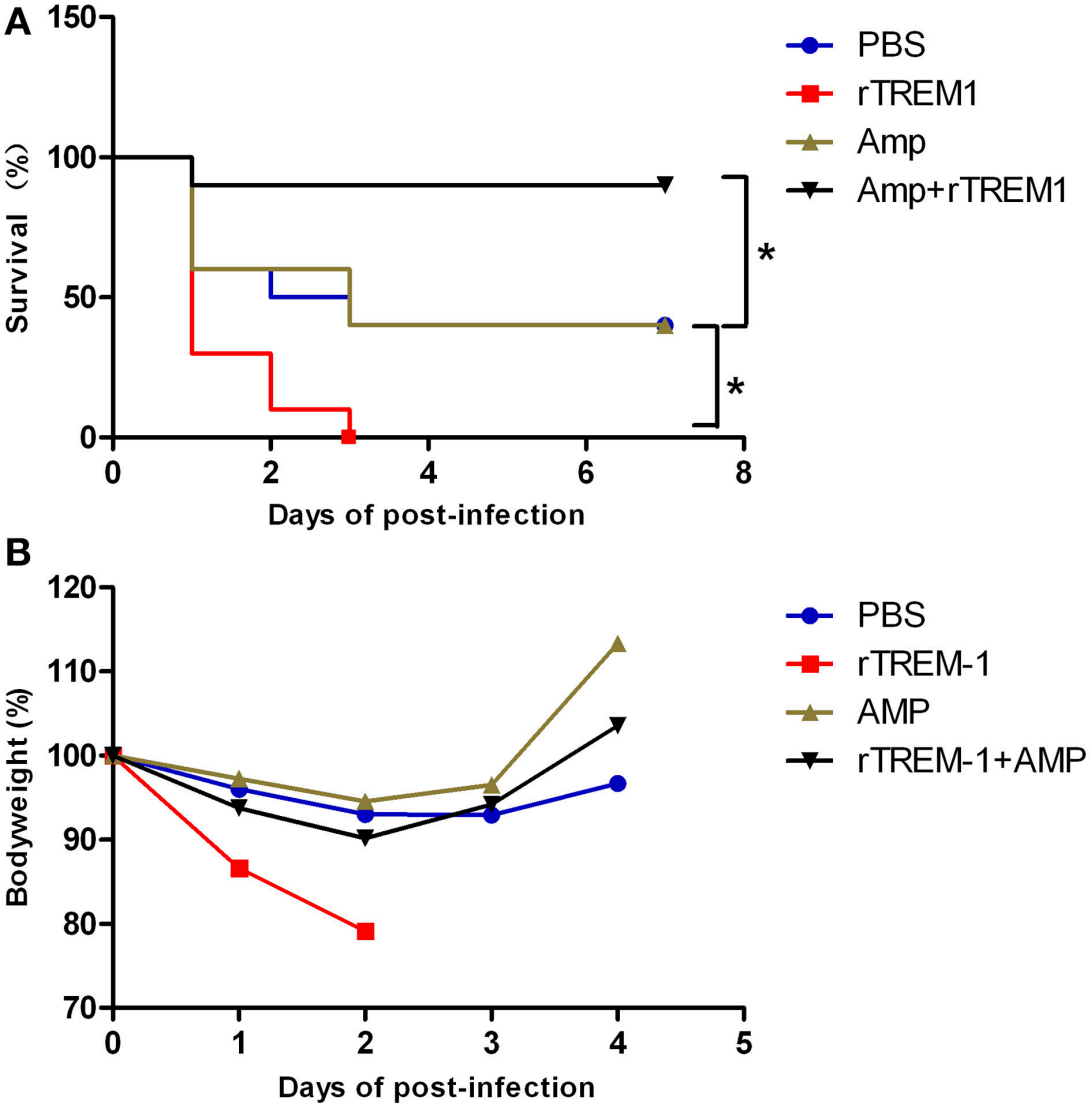
**Combined treatment with rTREM-1 and antibiotics protects mice against ***S. suis*** infection**. **(A)** Survival of mice infected with a highly virulent *S. suis* strain. The mice were treated with PBS, rTREM-1, antibiotics, or antibiotics+rTREM-1 at 3 h post-infection. **(B)** The daily body weight of mice infected with highly virulent *S. suis*. “^*^” represents *p* < 0.05 for the selected two groups.

In the present study, mice infected with *S. suis* showed sudden death and high fatality, similar to previous studies (Dominguez-Punaro et al., 2007; Dominguez-Punaro Mde et al., 2008; Ye et al., 2009). As described before, treatment with rTREM-1 alone intensified rather than reducing the severity of the clinical signs, which was also reflected by a change in body weight on days 1 and 2 post-infection (Figure 1). Furthermore, all of the mice treated with rTREM-1 alone died within 3 days, while 40% of the mice treated with PBS recovered from the infection (Figure 1). These data indicated that treatment with rTREM-1 alone cannot be used as a treatment strategy for STSLS (Bouchon et al., 2001; Gibot et al., 2004; Wang et al., 2012).

Antibiotic treatment is typically administered during the bacterial infection, although the treatment effectiveness on *S. suis* infection remains controversial. In the present study, treatment with ampicillin at a given concentration could improve the clinical manifestation in infected mice, which was reflected by a quick recovery of body weight (Figure 1). However, 60% of mice died during the infection despite receiving antibiotic treatment (Figure 1), which was similar to the outcomes of the clinical treatment of pigs and humans infected with highly virulent *S. suis*. In contrast, 90% of mice that received a combination of ampicillin and rTREM-1 recovered from the infection (Figure 1). These data indicated that rTREM-1 acted synergistically with the antibiotics to protect the mice against highly virulent *S. suis* infection.

**Figure 2:**
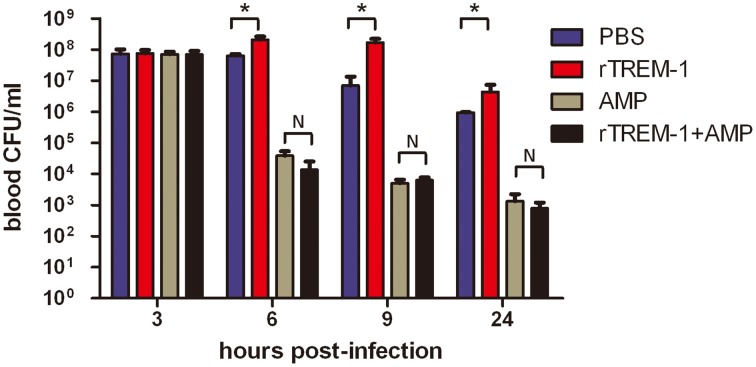
**The kinetics of bacterial clearance from the blood of mice**. Mice were infected with a highly virulent *S. suis* strain and treated with PBS, rTREM-1, antibiotics, or antibiotics+rTREM-1 at 3 h post-infection, and the colony forming units (CFU) in blood were compared at 6, 9, and 24 h post-infection. “^*^” represents *p* < 0.05 for the two selected groups, and “N” represents no significant difference for the selected groups at the given time-point.

Because the TREM-1-mediated inflammatory response was essential for *S. suis* clearance, the blockage of the signaling by rTREM-1 would cause a high burden of bacterial load, as described in our recent study (Figure 2). Undoubtedly, administration of ampicillin could effectively control bacterial growth when delivered with or without rTREM-1 (Figure 2). However, inhibition of bacterial propagation alone cannot significantly improve survival rates (Figure 1). In contrast, blocking the TREM-1-mediated inflammatory response and killing the bacteria at the same time had a significant effect against *S. suis* infection.

**Figure 3:**
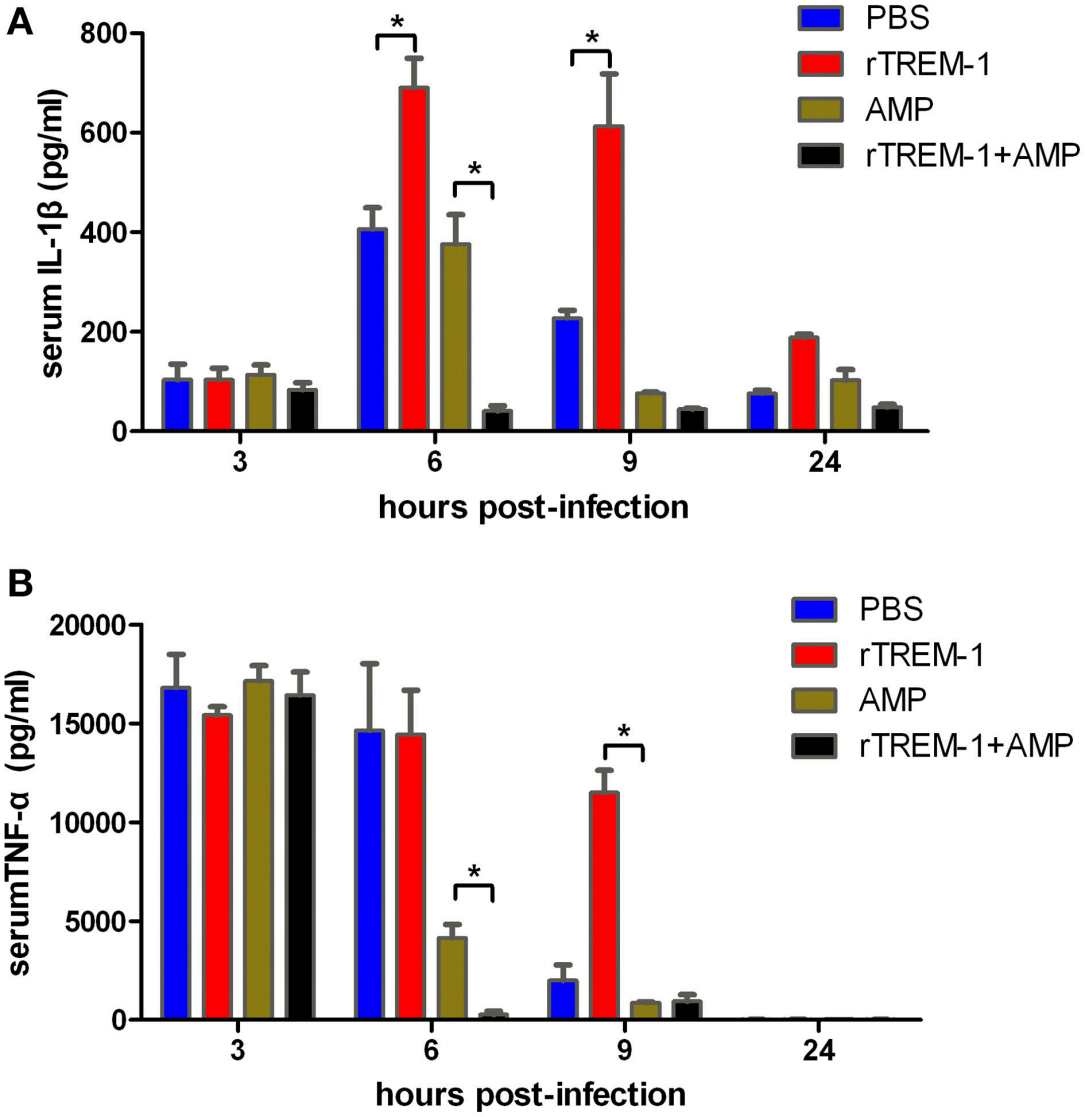
**Kinetics of serum IL-1β (A) and TNF-α (B) in mice**. Mice were infected with a highly virulent *S. suis* strain and then treated with PBS, rTREM-1, antibiotics, and antibiotics+rTREM-1 at 3 h post-infection, and the serum TNF-α and IL-1β were determined with commercial ELISA kits. “^*^” represents *p* < 0.05 for the two selected groups at the given time-point.

Highly virulent *S. suis* can rapidly induce high levels of inflammatory cytokines, resulting in a high fatality rate (Tang et al., 2006; Dominguez-Punaro et al., 2007; Dominguez-Punaro Mde et al., 2008; Ye et al., 2009). The correlation of severe inflammation and a high mortality rate was further supported in this study. Serum levels of IL-1β and TNF-α reached the highest levels 3–6 h post-infection before decreasing. The mice treated with rTREM-1 maintained high levels of pro-inflammatory cytokines even 9 h after infection (Figure 3). Treatment with antibiotics alone could promote bacterial clearance and then reduce the pro-inflammatory cytokine levels. However, the levels of IL-1β and TNF-α in mice treated with both ampicillin and rTREM-1 decreased very quickly (Figure 3), which indicated that treatment with antibiotics alone was not enough to rapidly reduce inflammation. This also indicated that enhancing bacterial death by ampicillin and blockage of TREM-1-mediated response by rTREM-1 at the same time could effectively protect the host against epidemic *S. suis* infection.

**Figure 4:**
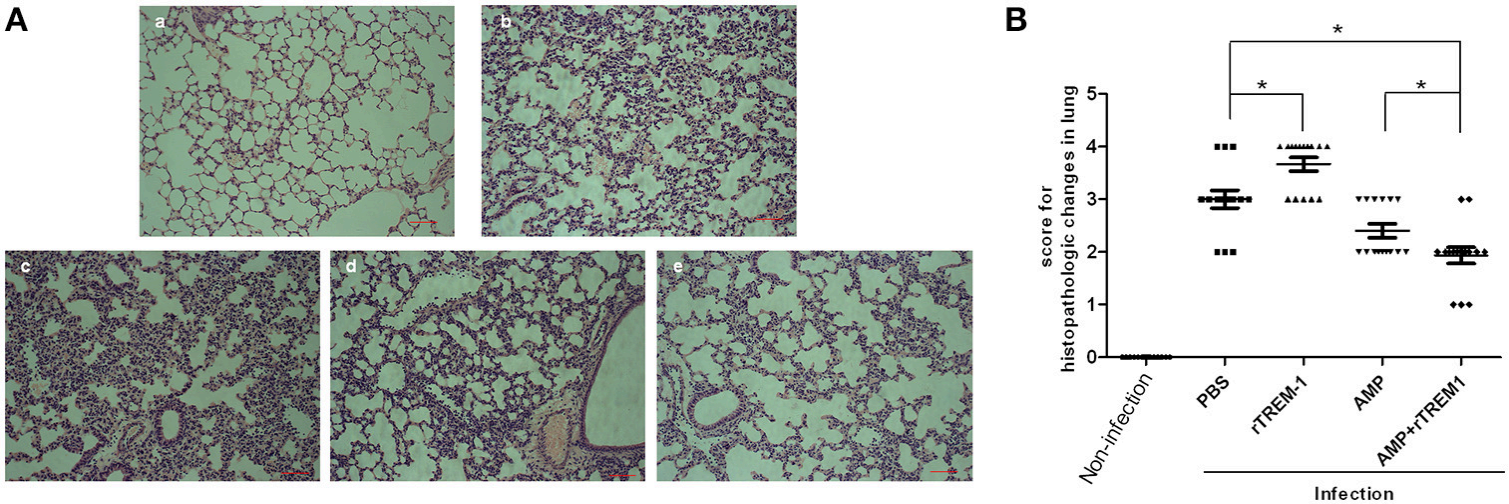
**The histopathological study on lungs of mice infected with highly virulent ***S. suis*** strain for 6 h with different treatment**. **(A)** The histopathologic evaluation of lung of mice treated with PBS (b), rTREM-1(c), ampicillin (d), and a combination of ampicillin and rTREM-1 (e) at 6 h post infection with highly virulent *S. suis* strain. The mock infected mice were served as a control (a). The red line represents 20 μm. **(B)** The comparasion of histopathologic changes of lung of mice infected with highly virulent *S. suis* strain with different treatment. Five areas were randomly selected from the every histopathologic section and then were scored based on the severity and inflammation. Scores 0, 1, 2, 3, and 4 represent normal, minimal, mild, moderate, and severe histopathologic changes. “^*^” represents *p* < 0.05 for the two selected groups.

The histopathological study of the lungs of mice infected with a highly virulent strain for 6 h also indicated that treatment with rTREM-1 could cause more severe lung inflammation in comparison with PBS treatment (Figure 4). The treatment with ampicillin alone alleviated the inflammation slightly, while treatment with both ampicillin and rTREM-1 could alleviate the inflammation significantly (Figure 4). These data indicated that the TREM-1-mediated inflammatory response directly contributed to STSLS in addition to contributing to *S. suis* clearance.

Until now, TREM-1 was an attractive target for the treatment of septic shock (Gibot et al., 2006, 2007), sepsis (Bouchon et al., 2001; Gibot et al., 2004; Wang et al., 2012; Pieters et al., 2013; Van Bremen et al., 2013), inflammatory bowel disease (Holden et al., 2009; Genua et al., 2014) and chronic inflammatory disorders (Schenk et al., 2007), autoimmune arthritis (Murakami et al., 2009), corneal inflammation (Wu et al., 2011), and hepatocellular chronic inflammation (Wu et al., 2012). However, in addition to TREM-1 contributing to inflammation, TREM-1 also plays a critical role in the clearance of *Pseudomonas aeruginosa* (Klesney-Tait et al., 2013), pneumococci (Hommes et al., 2014), *Kelbsiella pneumoniae* (Lin et al., 2014) and the highly virulent *S. suis* (Yang et al., 2015). This might be the reason to explain that blocking TREM-1 signaling alone cannot rescue the host from these bacterial infections. In the present study, we found that blocking TREM-1 signaling in the presence of antimicrobials could significantly improve the survival of mice against *S. suis* infection by efficient bacterial clearance and alleviated severe inflammation. These results could further promoted the researchers to evaluate the effect of blockage of TREM-1 signaling in the presence of antimicrobials against severe inflammation induced by *Pseudomonas aeruginosa, pneumococci*, and *Kelbsiella pneumoniae*.

## Author contributions

The experiments were performed mainly by CY, JZ, and LL, and some experiments were performed with the help of SP and LF. CY, LH, and AZ performed the data analysis with the help of RZ. Some experiments were performed according to the suggestion of MJ. The study was designed by AZ.

### Conflict of interest statement

The authors declare that the research was conducted in the absence of any commercial or financial relationships that could be construed as a potential conflict of interest.
